# Developing an effective and comprehensive communication curriculum for undergraduate medical education in Poland – the review and recommendations

**DOI:** 10.1186/s12909-023-04533-5

**Published:** 2023-09-07

**Authors:** Martyna Borowczyk, Agata Stalmach-Przygoda, Antonina Doroszewska, Maria Libura, Marta Chojnacka-Kuraś, Łukasz Małecki, Zbigniew Kowalski, Aldona K. Jankowska

**Affiliations:** 1 Department of Medical Simulation, Poznan University of Medical Sciences, Poznań, Poland; 2 Department of Medical Education, Center for Innovative Medical Education, Jagiellonian University Medical College Kraków, Kraków, Poland; 3 Department of Medical Communication, Medical University of Warsaw, Litewska 16 Street, Warszawa, 00-575 Poland; 4 Department of Medical Education and Simulation of Collegium Medicum, University of Warmia and Mazury, Olsztyn, Poland; 5 Institute of Polish Language, University of Warsaw, Warszawa, Poland; 6 Komunikacja z Pacjentem.pl, Warszawa, Poland; 7 Laboratory for Social Medicine, Ludwik Rydygier Collegium Medicum in Bydgoszcz, Nicolaus Copernicus University in Torun, Bydgoszcz, Poland

**Keywords:** Assessment, Clinical communication, Curriculum, Medical communication, Medical education

## Abstract

**Background:**

The recognition of the importance of effective communication in the healthcare system has been growing. Given that communication courses must be adjusted to the specificity of a particular culture, language, and other contextual issues, many countries and communities sharing a common language have proposed their recommendations for a communication curriculum for undergraduate medical education. To date, no recommendations have been developed for either any Central and Eastern Europe countries or for regions where Slavic languages are spoken. Their specificity of post-communist transformation should be acknowledged. This study aims to review communication curriculums and offer recommendations for medical communication training for undergraduate medical students in Poland.

**Methods:**

The recommendations were developed through an iterative consultation process with lecturers, faculty members of medical schools, and education coordinators. PubMed and Google Scholar databases were searched to identify full text English and Polish language articles on communication curriculum for undergraduate medical education. Additionally, the new Regulation of the Polish Minister of Science and Higher Education, defining educational standards for undergraduate medical education was analysed in search of learning outcomes that could be applied in communication skills teaching. The authors extracted the most relevant communication skill competencies, as determined by the process participants, discussed current challenges, including those of the COVID-19 pandemic era, and indicated best practices.

**Results:**

A review was conducted, and a set of recommendations was developed pertaining to the scope and methodology of teaching communication skills. The study included: (1) definition, (2) education content, (3) learning outcomes, (4) the recommended teaching methods. The recommendations are in concord with the graduate profile, as well as the current structure of medical studies. The authors listed and discussed the basic communication competencies expected of medical graduates, as well as medical communication course content viewed from different perspectives, including clinical, psychological, sociological, legal, and linguistic.

**Conclusions:**

Detailed recommendations aimed at integrating best practices into a comprehensive communication curriculum may promote successful teaching, learning, and assessment of medical communication.

## Background

The communication skills of healthcare workers are part of basic clinical skills [1]. Communication is a necessary tool for physicians that is of fundamental value during all stages of the treatment process [2]: from establishing the correct diagnosis [3] to the effective use of therapy available for a given patient [4].

With the increasing recognition of the importance of medical communication, its teaching has been incorporated into the core curriculum of undergraduate medical education. Medical faculties worldwide started to introduce appropriate courses to their curricula at the end of the 20th century. One of the first experiences came from the University of Lagos in 1984 [5]. More recent examples include Leipzig University Medical School (COMSKIL Communication Skills Training [6] and Longitudinal Communication Curriculum [7]), Charité - Universitätsmedizin Berlin [8], Ghent University [8], and Harvard Medical School [9].

The need for standardized teaching practices lead to several countries developing their own consensus statements. One of the first consensuses on the teaching and assessment of communication skills in medical schools came from Canada in 1992 [10] and 1993 [11]. One of the most influential was the UK consensus statement on the content of communication curricula in undergraduate medical education, first published in 2008 [12], and updated 10 years later [13]. Other notable national recommendations have come from India [14], Brazil [15], Korea [16], Germany [17], the Netherlands [18], and Switzerland [19].

Attempts have been made to offer international recommendations or consensuses based on the languages spoken, such as the Latin American, Portuguese and Spanish consensus on a core communication curriculum for undergraduate medical education [20] and the Basel consensus statement for German-speaking students [21]. One hundred twenty-one communication experts from fifteen professional fields and sixteen European countries have previously prepared a European consensus on learning objectives for a core communication curriculum devoted to health care professionals [22], as outlined by the goals of the Bologna Process.

Unlike the countries of Western Europe and North America [60], in Poland the introduction of the study of communication in the field of medicine was not implemented by faculties of medicine into the curriculum until the second decade of the 21st century. Scholars involved in developing communication curricula were able to draw on the experience of universities from Western Europe and North America. However, they had to take into account the historically, socially and culturally conditioned context of teaching in Poland.

Some medical educators believe that communication skills are constant across different cultures and languages. Also, UK communication skills curricula suggested that they can be implemented worldwide. However, other researchers question this assumption; Mirza argued that such skills cannot plausibly be considered universal [23]. She suggested several adaptations to the ‘communication curriculum wheel’ were needed before it could be utilized in a local context. She also pointed out that the core tasks of clinical communication must be learned and practiced in every context. However, applying those universal skills should be adapted to fit into a local context. Local culture, religion, structure of society, legal and ethical issues should be taken into account, as these profoundly affect all aspects of communication.

Also, the context of local language significantly impacts medical communication, which was reflected in the attempts to write consensus based not on the language spoken. Therefore, it became vital to prepare recommendations that were specific to the system of undergraduate medical education in Poland, including present day challenges.

Over the last years, Poland has undergone vast political and economic changes beginning with the postwar period, through the time of communism, political transformation, to capitalism and inclusion in the European Union. These political changes were accompanied by fluctuations in living standards and social inequalities [24].

Social transformations in the countries of North America and Western Europe have been spread over the years after World War II until today. The same direction of social processes in Poland (the same as in many other Central and Eastern European countries) appeared only after 1989 and brought dynamic (revolutionary) changes in Polish society (towards civil society).

It brought social differentiation and a revolution in attitudes. The transformation in 1989 affected many aspects of the functioning of Poles (economic, professional, educational).

Studies focusing on transformations in post-socialist countries have usually dealt with major economic and political shifts, particularly regarding democratization, civil participation, and the transition from formerly planned economies into market economies [25, 26]. Social inequalities emerged.

On the other hand, attitudes of Poles have also changed - those towards health itself (health awareness, health literacy) and – consequently - patients’ expectations in health care [27–29]. All this influenced the wide differentiation of the doctor-patient relationship visible today (from paternalism to consumerism). Findings reveal specific differences in doctor–patient communication in post- communist countries as a consequence of differences in trust and ambivalent relationship toward authority [30–35]. Diversification of education, health awareness, health competence, socio-economic status, but also different expectations of the patient towards the doctor requires taking these components into account in the recommendations for teaching medical communication.

The aim of the study was to review medical communication curriculum.

research and present recommendations for communication skills training for undergraduate medical students in Poland acknowledging their global and local context.Therefore, the aim was to prepare a curriculum including parts present in most published consensus that are global in nature, but also parts that take into account cultural differences and local language. The latters requires the teaching and learning to be targeted to central and eastern European countries. They need to be a reason for the unique parts of a medical communication curriculum.

## Methods

The recommendations for the undergraduate medical communication curriculum were developed through an iterative consultation process with 8 teachers, members of medical faculties, and the education coordinators representing the five Polish medical schools (Bydgoszcz, Kraków, Olsztyn, Poznań, Warszawa), all of whom are members of the Polish Society of Medical Communication.

For the study, a narrative literature review was performed using Web of Science, Google Scholar and PubMed/MEDLINE databases. The review followed previous guidelines on the development of a narrative review outlined by Dixon-Woods et al. [36] and Peters et al. [37]. The search strategy included Medical Subject Headings terms and keywords: “communication and (“medical education” or “medicine”) and (“curriculum” or “undergraduate”)”. Reference lists of all the selected articles, previous reviews, and meta-analyses were hand-searched for any additional articles.

The narrative discussion was aligned with individual articles and interpretations of relevant articles by organizing it into sections: (1) definition, (2) education content, (3) learning outcomes, (4) the recommended teaching methods.

The inclusion criteria were full text articles published in English and Polish between January 2001 up to December 2022 concerning communication curriculum for undergraduate medical education.

One author selected papers which fulfilled the inclusion criteria and extracted data for the outcomes using a standardized data extraction form. Papers not describing learning outcomes concerning communication skills were excluded. Another author rechecked the extracted data. Out of 6381 papers chosen by the search strategy including Medical Subject Headings terms and keywords, 168 were included after reading the titles. Then, a total of 73 were included after reading the abstract. Eventually, after reading the full texts 28 articles were included to the final analyses based on the quality of the studies.

The review process was used to list learning outcomes concerning communication skills [38]. The authors identified common communication skill competencies by comparing the Calgary Cambridge Observation Guides [39], Kalamazoo Consensus Statement [40], the Four Habits Model [41], The SEGUE Framework [42], and previous communication skills lists that the authors have used.

The new Regulation of the Minister of Science and Higher Education on educational standards for undergraduate medical education [62] also underwent a content analysis in order to select learning outcomes including communications skills. Additionally, the authors discussed present day challenges posed by the COVID-19 pandemic.

To develop the communication curriculum recommendations for undergraduate medical education in Poland and to achieve a consensus, a systematic academic approach and the Delphi technique were used. The process of the curriculum development was split in 6 phases within the process: (1) development of a list of learning outcomes concerning communication skills on the basis of literature, existing consensus statements, frameworks and guidelines, (2) in person expert meeting to discuss: (a) general communication competencies, (b) education content based on various aspects of communication, (c) educational content concerning medical consultations, (d) the recommended teaching methods, (3) development of a first draft of recommendations, (4) online meetings and electronic discussion process to revise the first draft; (5) second Delphi round to test the revised draft and make suggestions for improvement (6) a final Delphi round for the recommendations.

The objectives and learning outcomes needed to be most comprehensive in inclusion and detailed. They were defined according to Bloom’s taxonomy using a skills-based approach. Ambiguous aspects among experts were reworded and new aspects incorporated.

## Results

The study includes: (1) definition of medical communication, (2) education content, (3) learning outcomes, (4) the recommended teaching methods.

### Definition

Medical communication is an interaction between healthcare professionals (doctors, nurses, midwives, etc.) and patients (and their relatives) [43], as well as the interaction between healthcare professionals themselves (interprofessional communication in medical teams) [44], taking place as part of a healthcare relationship [45] within a treatment or a prophylaxis. Most often, it takes the form of a face-to-face encounter [46], but various means of remote communication are also possible. The overall goal of such communication is to provide the best healthcare.

Medical communication has also become a research discipline, which has been developing dynamically (including in Poland) for over a dozen years, as evidenced by the growing number of studies and publications. Research and medical education need to go hand in hand for best practices to be gradually institutionalized [47].

### Learning outcomes

Communication competencies are necessary to make medical communication effective. Those competencies comprise knowledge (appropriate background), skills [48], and attitudes [49] that lead to an understanding between medical staff and the patient (and their relatives) [50, 51] and between the medical team members themselves. This understanding considers the full context.

The process of medical communication remains strictly linked with general communication competencies. Those competencies used at different stages of the consultation contain:

- communication skills required to build a relationship with the patient.
- verbal and non-verbal communication in the relationship between the doctor and the patient.
- communication skills in telemedicine.
- active and careful listening - methods to improve communication.
- empathy and cognitive empathy (including clinical empathy) and their importance in medical communication.
- overcoming communication barriers.
- assertiveness in communication.

With the proper training every graduate should be able to:

- recognize the patient’s perspective (their beliefs about disease, their expectations and fears), effectively connect it with the biomedical perspective during the information gathering stage, and provide information in an understandable manner;
- easily interact with the patient and his relatives using various channels and forms of communication, during both the diagnostic stage and throughout the therapeutic process;
- build a trusted relationship with the patient based on the mutual understanding of the role and course of action (i.e., caring for the maintenance and development of the patient’s well-being and health in collaboration with doctor);
- make medical decisions together with the patient while respecting their autonomy;
- serve as the patient’s advisor and guide throughout the diagnostic and therapeutic processes;
- skillfully and consciously shape a relationship with the patient, even during periods of increased stress and emotional tension;
- behave in a mature and empathetic way; knowledge and skills allow him to flexibly interact with various patients (and in different situations);
- have comprehensive medical knowledge, extended with elements other fields (including psychology and sociology), he gives patients multidimensional help in the context of therapy as well as prevention and strengthening health or improving functioning, including environmental interventions;
- demonstrate the ability to absorb new knowledge and skills and adjusting them to changing working conditions and requirements;
- care about the personal well-being (sets the realistic goals and has requirements to each other, adequate to their possibilities), because the students know that the smooth functioning of them as doctors and the whole team they are a part of depends on it;
- communicate efficiently with members of the medical team and manages conflict situations.

### Education content

A proposed content list to be included as a part of the communication curriculum in medicine and dentistry may ensure consistency and a high and similar level of education.

The listed issues relate to general problems (introducing the subject of medical communication and exploring it from different perspectives) and detailed (related to specific situations in doctor’s work). All the topics listed are directly related to the practice of medicine.

Although general interpersonal communication competencies remain a basis for medical communication, various aspects of communication should be considered, such as: psychological, sociological, legal, ethical, and linguistic.

**Fig. 1:**
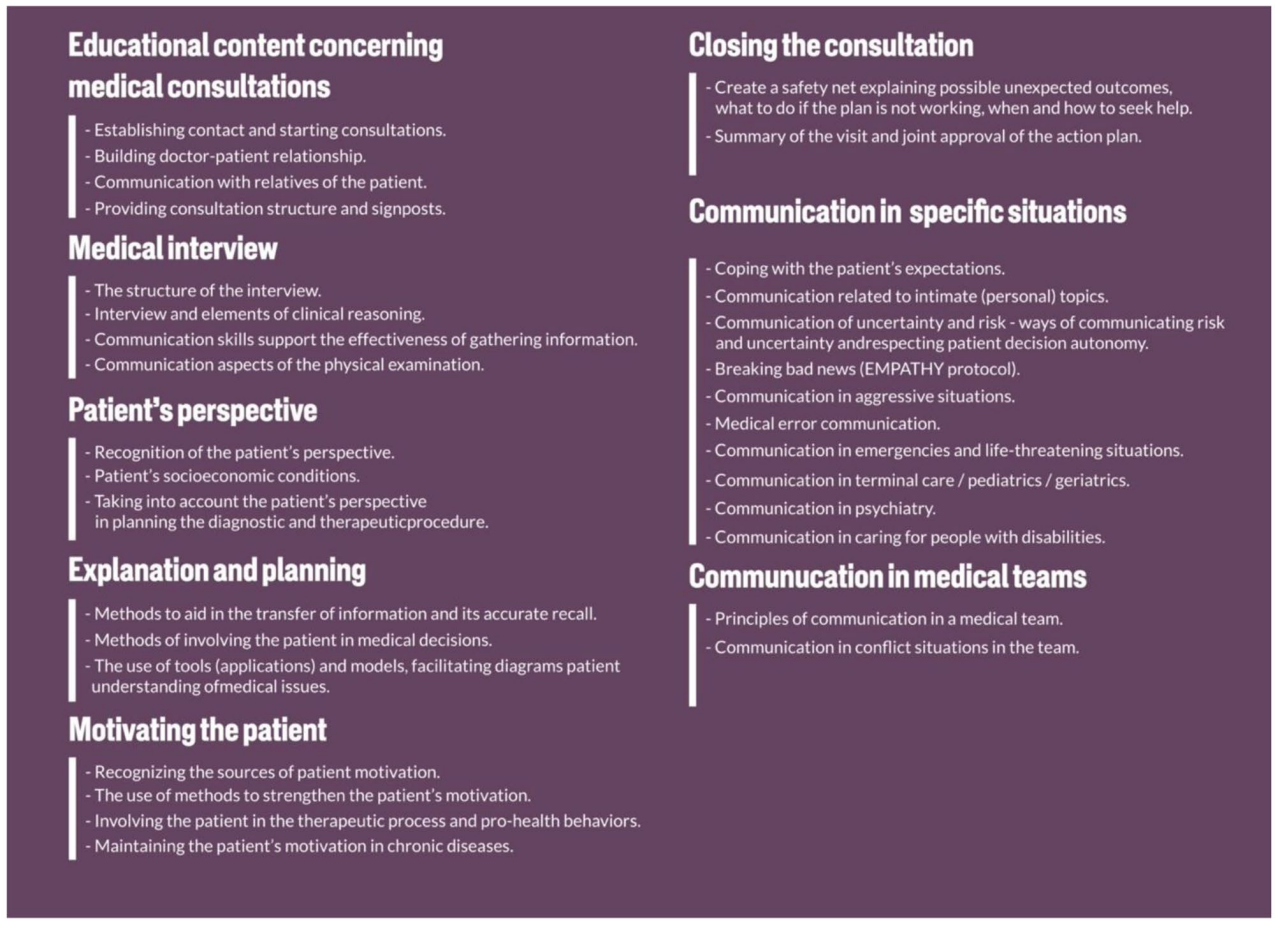
Educational content concerning medical consultations

Medical consultation may be considered as a core situation in medicine. Figure 1 describes educational content concerning it.

Education content should include communication in specific situations:

- Communication in terminal care - conversation about the end of life, values important to the patient.
- Communication in pediatrics - communication with patients of different ages - infants, preschool children, early school children, teenagers. Communication in the triad - doctor - patient - patient caregiver.
- Communication in geriatrics - conversations with elderly patients, people with dementia, with cognitive disorders or degenerative diseases e.g. Alzheimer disease. Communication in the doctor-patient-companion triad.
- Communication in psychiatry - communication in the situation of exacerbation of mental illness, communication with patients with mental disorders, respect for the rights of patients with mental disorders.
- Communication in caring for people with disabilities - taking into account the special needs of patients with disabilities, especially intellectual disabilities, Deaf, visually impaired and blind patients.

Issues related to motivating patients are presented as a separate module in teaching communication in medicine for several reasons [53–55]. First, motivating patients requires specialized knowledge and skills, from the phenomenon of motivation itself, the mechanisms affecting motivation, the psychoneurology of habit change, patients’ individual functioning styles and how to motivate adherence based on this knowledge. Second, motivating patients can also be a challenge due to personal and family situations. Creating a therapeutic alliance while respecting the patient’s autonomy, showing support but also soliciting or encouraging supportive relationships is a scope that goes beyond standard communication mechanisms. Third, motivating patients also requires health care professionals to have a high degree of self-awareness and emotional maturity, which in turn implies the ability to distribute responsibility appropriately, avoiding pitfalls and mistakes along the way to share responsibility for achieving therapy outcomes, without engaging in emotional games with patients or their family members.

### Teaching methodology

The recommended teaching methods are grouped into types as presented in Table 1. The order of the methods is not based on their hierarchy or evaluation. Various kinds of teaching methods work well for different content and at different stages of education. For example, lecture methods allow students to be introduced to the theory of the problem. Problem-based learning in small groups allows them to independently identify difficulties in a given situation and look for solutions. During conversations with a simulated patient, students have a chance to practice and verify the principles of good communication and develop specific skills (before meeting real patients). Close reading of literary texts and their interpretation in a group strengthens openness to patient narratives and awakens empathy in future doctors. It is worth keeping all these methods in mind and using them together, depending on the needs and abilities of students.

**Table 1:** The recommended teaching methods

	TYPE OF TEACHING METHOD			
	PROBLEM	FEEDING	VALORISATION	PRACTICAL
Examples of techniques	Case study	Lecture	Roleplaying	Simulation - conversation with a simulated patient, overview and feedback
	Problem-based learning	Presentation	Visualization	Writing scenarios of interaction with the patient
	Movie	Anecdote	Careful reading (of literary texts and narratives about the disease and disability)	Conversations with patient avatars (using artificial intelligence)
	Didactic games	Story	Reflective discussion	Conversations with real patients
	Discussion		Reflective writing	
	Brainstorm Active’ small group work facilitated lecture			Multidisciplinary group learning (medicine, nursing, pharmacy)
	Correcting conversation scenarios			
	Learner self-directed learning			

## Discussion

The learning outcomes of these proposals provide guidance to the universal introduction, support, and development of a communication curricula as a part of undergraduate medical studies in Poland.

The following set of recommendations is the first of its kind in Central and Eastern Europe. Country and language-orientated list of content, learning outcomes and teaching methods may inspire experts from other countries in this region and encourage them to prepare recommendations on the development of communication skills in medicine that correspond to Slavic countries’ linguistic and cultural contexts.

### Definition

The reference for the definition of communication in medicine is the one formulated by Jan Doroszewski in 2007 [43]. Its basic assumptions have been maintained and the essential determinants of medical communication from the perspective of the current state of knowledge and practical experience have been updated.

The importance of communication in clinical practice has also been recognized in Poland [56–58], as attested to by changes in the medical education curriculum, especially at medical and medical-dental faculties. Medical universities and faculties are making efforts to incorporate communication studies more thoroughly and effectively into their curricula [59], in order to equip future healthcare professionals with the necessary occupational competencies. For this process to be successful, best practices need to be identified and be implemented in a culturally sensitive fashion. With the growing demand for communication skills training to be incorporated into undergraduate medical education, there is a need for the standardization of teaching practices, and the continued assessment of communication skills.

This history of teaching communication in healthcare in Poland justifies the need for a recommendation [61]. Contemporary sociological and cultural changes in Poland make recommendations consistent with them necessary. Until recently, communication classes in Poland were optional for medical students. However, it does not mean that the topic of communication was totally absent from the curriculum. For example, interviewing skills were incorporated in a non-standardized (and undocumented) fashion into clinical and humanities subjects. The only point of reference regarding course content is the Regulation of the Minister of Science and Higher Education [62], which indicates the learning outcomes that a student of medicine must attain. Some of these outcomes relate to knowledge and communication skills. However, these effects are described in general terms, allowing for great freedom in interpreting the scope of topics and teaching methodology. The number of learning outcomes and their formula are limited. What is lacking is a better description of the content of communication skills teaching, the way of teaching communication, its methodology, and the competencies of communication skills educators. This leads to a situation where communication becomes an obligatory part of education but remains completely unstructured. The various curricula differ not only in the number of hours devoted to the topic of effective communication, but also in the type of classes (lectures, seminars, simulations), in the number of years of study, and in the staff tasked with developing the communication skills of the students. Due to the general nature of the learning outcomes set out in the regulation, there are also differences in the educational content. The Polish Society of Medical Communication’s recommendations are a proposal related to the organization of both the method of instruction and the subject matter, in order to help build a uniform communication curriculum at all medical universities.

### General communication competencies

The perception of communication should be interdisciplinary. Communication as a field of study and competence draws from various sciences. Integrating this knowledge and skills together is essential. As a result, this leads to the acquisition of competencies that the graduate can use in clinical practice. Therefore, the first part of the educational content (Fig. 1) is related to students’ understanding and acquiring knowledge and skills in various fields. Later in education, these elements are integrated with the clinical context.

**Fig. 2:**
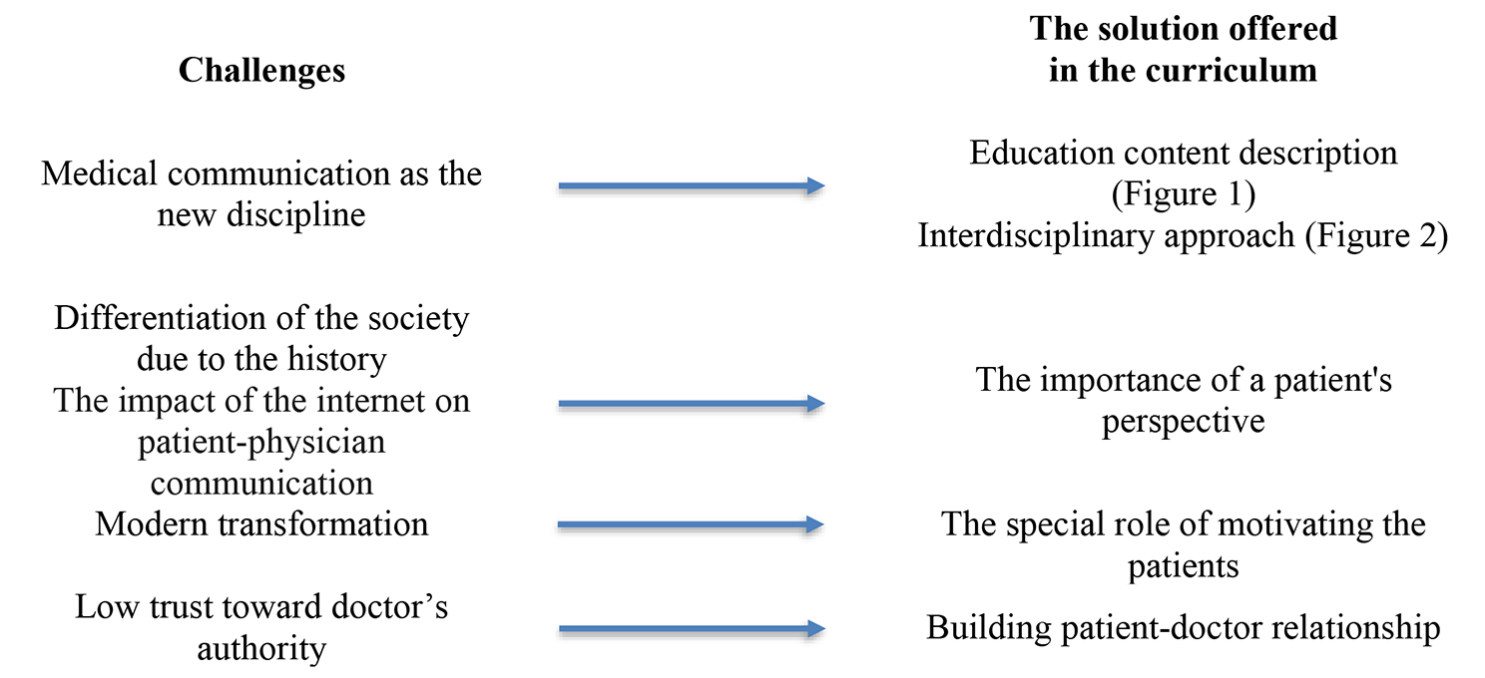
Implications for Polish and Eastern European Medical School

Psychological, sociological, legal, ethical, and linguistic aspects of communication have been detailed (Fig. 2).

The basic skills associated with the consultation stages are based on the Calgary Cambridge model [63, 64], which accurately describes them. This list has been expanded with motivation, as it is vital in numerous diseases of affluence and as it should counteract the very poor health literacy of Polish society. Also, the patient’s perspective should be emphasized due to the still strong paternalism in Polish healthcare.

Teaching methods should be selected according to the aims and means of instruction. Using practical methods is highly recommended.

### Why do communication skills continue to be important?

Multiple studies confirm that the manner in which a physician communicates with his or her patient directly impacts patient satisfaction [65, 66] and measurable treatment outcomes [67]. Quality communication also counteracts the effects of professional burnout among medical personnel [68, 69]. Additionally, the recognition of the importance of team communication is also growing [70] as it exerts influence on the effectiveness of the provision of health care [71]. Paradoxically, technical progress and the introduction of modern digital solutions have not diminished the role of interpersonal communication skills in the practice of medicine but have made them even more crucial. New challenges, such as the COVID-19 pandemic, have demonstrated that effective communication matters more than ever [5, 72].

The process of educating doctors depends on the medical culture [73] and evolves with a changing world [63]. Successive technological and digital revolutions (new diagnostic tools, incremental changes and developments, specialization of knowledge, computerization, telemedicine) and social and cultural changes (increased autonomy of patients, a growing number of chronically ill people) have influenced medical education [74]. Still, it affects the practice of medicine [47].

The practice of medicine requires current and future doctors to develop new clinical skills, demonstrate broadly defined competencies (including communication skills), and emotional maturity. One thing does not change: medicine must maintain its primary goal of providing support to others in need, and to help facilitate their return to good health in life-threatening situations.

Being a doctor is not only about providing medical services. It is most importantly having – the privilege of building a special relationship with another person based on trust, respect, and an openness that no machine can replace.

A model utilized to train doctors should meet various requirements and expectations [9]. When designing this model, various factors and trends should be considered. They will shape the future working conditions of doctors. Medical education should be a response to these challenges. To be responsible enough, the priorities of education at the stage of undergraduate education should already change and include key competencies in teaching (especially communication) that will allow doctors to practice their profession effectively. They will also support the smooth operation of the entire healthcare system.

Medical universities should follow modern standards of instruction with a view toward the future practice of medicine.

### The spiraling of teaching from the classroom to the wards

The development of highly competent communication skills should be initiated as early as possible, and optimally should be made a permanent part of the curriculum throughout all the years of a student’s medical studies. During undergraduate medical education, the student should initially develop knowledge and appropriate attitudes. They will transpose into simple skills, and over time into more complex ones.

The introduction of communication skills training should also be integrated as an educational tool into inpatient and outpatient settings, as well as in medical simulation centers. The curriculum should be structured in such a way as to enable students to acquire practical communication skills that are essential in the practice of medicine. For the best education of practical communication skills, the number of students included in the study groups should not exceed the number of students in the clinical training groups. In Polish medical universities, this means six-person student groups.

Some studies suggest that communication skills can diminish during the four years of medical school [9]. Which makes the creation of communication competence training programs an important necessity. Medical communication is allotted a limited amount of space in the curriculum. This curricular time must be expanded in order to enhance the important role of effective communication [75]. It is also important to continue communication skills training during the clinical years of undergraduate medical studies. To avoid the erosion of skills over time, clinical skills training should be continuous and supported by the institutional authorities [76].

The medical communication curriculum and classes should be designed, coordinated, and implemented (at least partially) by an internal unit responsible for the enhancement of communication competencies at a given university. Entrusting these tasks to a unit specialized in medical communication ensures that the consistency of the content taught in various classes will be maintained. Moreover, the appropriate preparation of those teaching communication subjects, and the employment of the appropriate didactic methods will ensure proper outcomes. The team responsible for teaching communication should employ specialists in medical communication who have certificates confirming their qualifications in this field. Communication teachers should be well prepared, both in terms of theoretical knowledge about communication, the necessary knowledge of medical practice and didactic competence. It is necessary to create training opportunities in this area. These factors should guarantee a high quality of education.

However, appropriate care should also cover teachers’ needs and prevention of their burnout. Medical faculty stress/burnout links directly to a willingness to implement medical school curriculum change [77]. Occupational burnout directly reduces the readiness to change. Therefore, to have successful academic reform in medical schools, it would be beneficial to assess and manage occupational burnout among clinical faculty members.

### Assessment methodology

Assessment of practical skills is crucial in the learning process and should be an integral part of the curriculum. Formative assessment and summative assessment [64] are recommended. The first is to verify and monitor the direction of development of current students’ knowledge and skills, and the second is to give a final grade for a given course, a task, or a block of tasks.

Both classic oral examinations (the so-called bedside examinations) and some written forms of examinations (such as SJT - Situational Judgement Tests) can assess communication skills [64]. However, they do so in a selective and unstructured manner. More structured methods of assessing clinical skills are OSCE (Objective Structured Clinical Examination) and mini-CEX (mini-Clinical Evaluation Exercise). OSCE is an exam where students in a simulated environment go through a series of time-limited stations where their clinical skills are assessed using standardized assessment methods (usually checklists) [78]. Communication skills could be assessed while stations dedicated to assessing specific communication skills (e.g. history taking or breaking bad news) or during stations testing other specific subject areas or domains [79]. It is worth emphasizing that the assessment of individual components of clinical communication encompasses the evaluation of both simple skills (such as the ability to ask appropriate questions, avoid jargon, and active listening) and more complex ones (such as using protocols for delivering bad news). Components related to verbal and nonverbal communication, as well as written and oral communication skills, may also be subject of the evaluation.

While the aforementioned components, referring to specific skills, can be assessed using well-consulted checklists, components associated with attitudinal aspects, such as empathetic approach, are better evaluated using a global rating scale.

One of the key elements enhancing OSCE standardization is the use of simulated patients (SP). SP is a person who is trained to present standardized scenarios, which ensures that all students are tested on the same clinical encounter [80]. This reduces variability and provides a consistent testing environment for all students. Additionally, SPs provide a safe environment to assess students without harm to real patients.

Another very frequently used standardized clinical skills exam is the mini-CEX. Unlike the OSCE, the mini-CEX (mini–Clinical Evaluation Exercise) is an exam from the workplace-based assessment (WBA) group and is conducted in a real environment in a hospital or outpatient clinic. The mini-CEX is a brief, focused assessment that can be completed within 10–15 min and is intended to be repeated multiple times over a learner’s training period to track their progress and provide ongoing feedback. Clinical communication is not only communication between the patient and HCP (healthcare practitioners), but also communication within the team so methods from Multiple-Source Feedback are recommended. Multiple-Source Feedback (MSF), also known as 360-degree feedback, is a formal questionnaire-based method of evaluating the performance of medical trainees that involves soliciting feedback from a variety of sources, including colleagues, patients, supervisors, and self-assessment.

Summarizing there is no single, best assessment method that can provide all the data required for assessment anything so complex as communication. It is important to note that assessing communication skills should be an ongoing process, with multiple opportunities for students (HCP) to practice and receive feedback throughout their medical education (work).

### Implications for polish and eastern European Medical School

As underlined in the background – the main argument for building Polish recommendations is a differentiation of the society due to the history of Poland and its modern transformation. Doctors meeting the patient will meet people of highly different expectations toward healthcare professionals, highly different health competencies, socio-economical status and preferable models of care (from paternalism preferred to consumerism) [27]. It makes the most important point to be acknowledged in thinking about local and global perspectives in preparing medical communication curriculum. What is needed is underlying the importance of a patient’s perspective in communication.

Specific for post-communist countries, ambivalent attitude towards authority means that on one hand one has to obey authority, and on the other, there is no trust in authority doing any good. In this regard, the basic ambivalence towards any kind of authority relates to the typical authoritarian figure, a doctor, then this ambivalence would come into play in physician-patient relationship. Trust towards doctors’ authority levels in western countries for some age groups tends to exceed 70% [30, 32, 35] whereas in post-communist countries trust is at the level of 21–22% [31, 33, 34]. This substantially means we have to assume that social desirability is an even bigger problem in post-communist countries than in western countries. Adherence might as well be a huge problem due to difficulties in addressing adherence. Patients manifest ambivalence towards authority when getting prescription, a drug or a recommendation of a certain change in behavior and sometimes they do not even confess that they have a problem not to be adherent. It is why within the education content concerning medical consultations, teachers should include motivation as an additional aspect to the Cambridge- Calgary approach. In an original model motivation is often described as a higher level communication skill and is understood as not required in every consultation, finding its place in special situations. It should have a different place in Polish, as well as other Central and Eastern Europe countries. Distrust seen in Polish society has an impact on a need to underline the role of building patient-doctor relationship in medical communication.

Instilled ambivalence towards authority plays also a crucial role in a way students are willing to accept feedback from seniors, who are authority. And this is why they seem unable and not willing to give feedback to each other. Preliminary data reveals problems students have with receiving or giving feedback. Reports from teachers say that students at the least indication of the slightest criticism in a feedback, become resistant, do not accept it, and instead of analyzing what has just been said, fight back using counter arguments, which brings the whole idea of feedback to the point of failure. Teachers should pay special attention to this point. It is also why there is a particular need for a good student-teacher relationship and friendly learning environment, as well as for teachers who care for themselves and are not burned out - to be attentive and convincing.

### The novelty of the curriculum in global and local context

By comparing previous medical models/frameworks of communication curriculum [1–23, 39–42, 63], missing domains of communication have been found that need to be added.

**Fig. 3:**
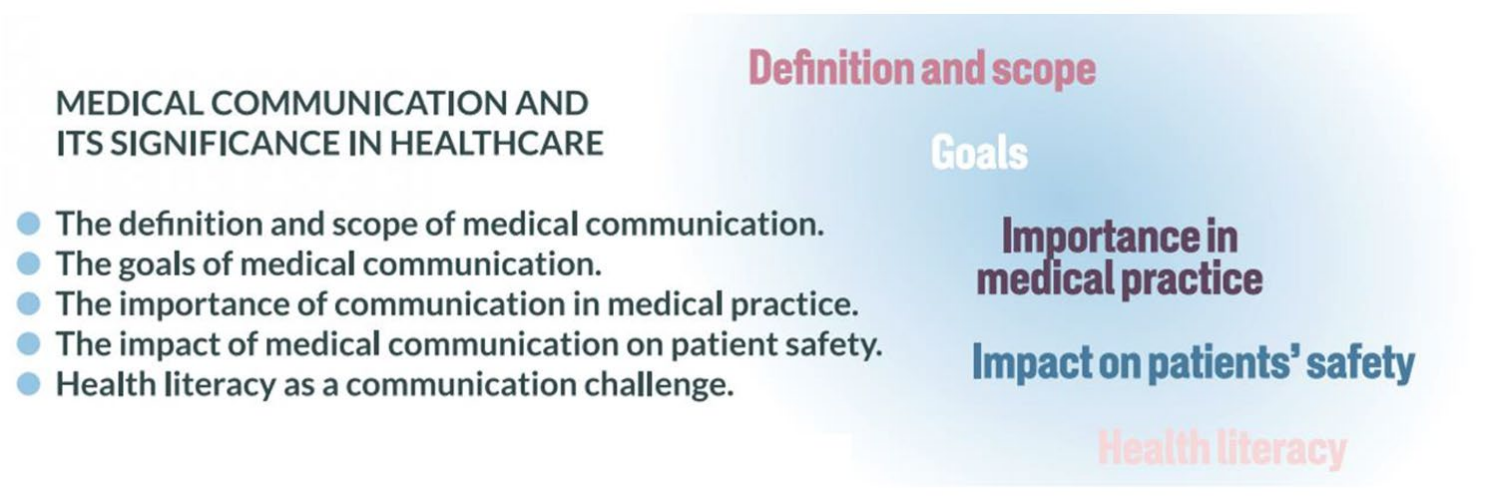
Education content (general)

Unlike educational content concerning medical consultations, which remains rather similar worldwide, education content describing medical communication and its significance in healthcare is characteristics for Polish and non-Western. In the local context – as described in our study – new definition and scope, goals, importance in medical practice, impact on patient’s safety and health literacy (Fig. 3) should be interpret in a context of language, history, geography, economics, and religion. In Poland and other non-Western country medical communication as a discipline has been developing lately with no traditions of teaching. Therefore, new context should be added. What is different than in other curriculums is also underlying of building doctor-patient relationship as an antidote for trust loss and authority’s crisis, present in the changing world of Poland and non-western countries. The role of patients’ motivating is particularly important in this curriculum, as teaching of communication should increase the efficacy of therapeutic process by patients’ better adherence. Other curriculums concentrate on sharing information. It remains a basis, but doctors should be able to understand patient’s motivation and shape it.

Polish school of medical communication share their own breaking bad news protocol (EMPATHY protocol) [52] (Emotions, Meeting, Patient’s perspective, Adequate language, Truth, Hope, Yes for patient’s empowerment).

The change that occurred after the creation of the models used so far is the widespread availability of the Internet, which is a game changer in doctor-patient communication.

The Internet is the first place people go to when they need information on medical topics. Searching for health information online has a strong impact on doctor-patient communication, presenting both advantages and challenges. Contemporary communication training must give students the ability to explore the patient’s perspective in relation to information obtained from various sources by the patient.

**Fig. 4:**
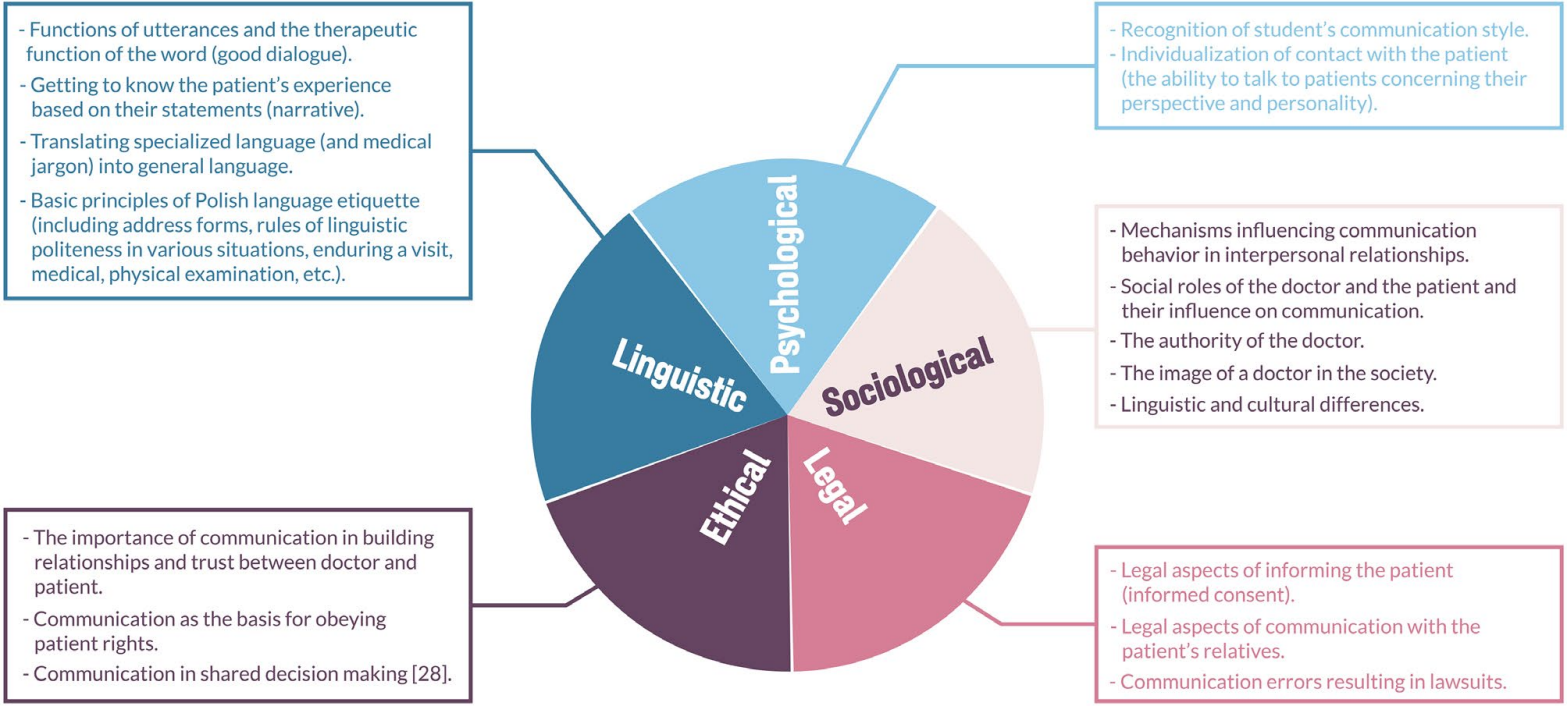
Education content based on various aspects of communication

Paying attention to the interdisciplinarity of communication (Fig. 4) is new and specific for Poland. Medical communication in Poland has many sources, it stems from the interest of researchers from various disciplines in these issues. As a result, communication competences in the Polish consensus are perceived in terms of socio-medical/medical-social competences.

This corresponds to the widely perceived competences that are considered the goal of modern medical education, and at the same time indicate the close connection of communication skills with knowledge from other disciplines that are necessary in practice. These elements appeared in the Polish consensus since the development of teaching communication is associated with intensive socio-economic and legal changes, and hence the need to take them into account.

### Future directions and challenges

Poland faces challenges and pitfalls regarding the teaching of effective medical communication. The most critical barriers today in this field include (1) incomplete understanding (also in the medical community) of separateness and interdisciplinary foundations of medical communication, (2) misidentification of communication with persuasive (or even manipulative) techniques, and (3) perception of communication competencies separately from clinical practice.

In order to optimize undergraduate communication skills teaching in practice, Junnod Perron cited the special need to: (1) modify the climate and structure of the working environment so that training and teaching of good communication skills in clinical practice become valued, supported, and rewarded; (2) extend communication skills training to all fields of medicine; (3) regularly provide structured training and adapt it to trainees’ needs [81].

There is a need for developing a communication skills training program as the communication skills of many students turned out to be inadequate [82].

However, the quality of delivering the course should be high. Researchers in India found a high prevalence of unfavorable attitudes toward communication skills classes based on a poor quality of the subject [83].

The passage of time and the added experience gained by teachers may promote change. Students’ attitudes toward learning clinical communication skills at the end of medical school have greatly improved over twelve years in two Norwegian medical schools from 2003 to 2015 [84].

Of course, competent communication includes an essential range of skills, and in order to maintain these skills beyond the undergraduate stage, continuing education will be needed [85].

Training in communication skills requires approaches which are different from the instruction related to other clinical subjects. It is also a challenge to ensure that students not only absorb the nuances of communication and interpersonal skills but adhere to them throughout their careers [14]. Due to the potential benefits of online learning, the educational adaptation of the recommendations in the form of online learning will be needed in the future as an alternative to maximizing the safety of all stakeholders and ensuring easy and timely access to learning materials and sessions.

The content of the recommendations and their method of building the student’s attitude is also applicable in the special situation of remote communication with patients. This communication requires the doctor to be aware and important of its individual elements. Therefore, the importance of teaching this competence will increase in the future.

Based on the experience of Polish and foreign medical universities, detailed recommendations for the organization and training of communication competencies on medical courses are offered to integrate views on teaching, learning, and assessment of clinical communication. It should be extended to and adapted to other medical faculties in the long term and adjusted to their specific needs and conditions.

It is hoped that this paper may be of assistance to those involved in the planning, development, application, and evaluation of medical communication curricula, especially new ones emerging in Central and Eastern Europe. Although designed for undergraduate education, the consensus statement provides a starting point for further professional development.

Changes in the practice of medicine should lead rapidly to adjustments in the curricula content.

The responsible and wise implementation of the teaching of medical communication at Polish universities is of great importance to the future of the healthcare system, the performance and efficiency of doctors and medical teams, and in the level of the quality of care that patients receive.

The necessary curriculum and organizational changes should be evolutionary, not revolutionary. It is essential that training in effective communication be coherent to the rest of the medical curriculum. Gradual integration of new techniques into the existing teaching model promises best chances of success. What is desired is also an approach change of the faculty members, who need to share the belief about the importance of medical communication teaching.

## Data Availability

The datasets used and/or analysed during the current study are available from the corresponding author on reasonable request.

## List of Abbreviations

COVID-19: Coronavirus disease
EMPATHY: Emotions, Meeting place, Patient’s perspective, Adequate language, Truth, Hope, Yes! (Patient’s empowerment)
HCP: Healthcare practitioner
Mini-CEX: Mini-Clinical Evaluation Exercise
MSF: Multiple-Source Feedback
OSCE: The Objective Structured Clinical Examination
SEGUE: Set the stage, Elicit information, Give information, Understand the patient’s perspective, and End the encounter
SJT: Situational Judgement Tests
SP: Simulated patient
UK: United Kingdom
WBA: Workplace-based assessment
